# Clinical activity and safety of nivolumab in combination with ipilimumab in metastatic melanoma: findings from REALIPINIVO, a real-world study

**DOI:** 10.3389/fimmu.2026.1738772

**Published:** 2026-04-16

**Authors:** Francesco Caraglia, Giuseppe Argenziano, Marcella Scala, Francesca Sparano, Teresa Del Giudice, Maria Chiara Sergi, Antonio Maria Grimaldi, Miriam Forte, Silvana Cozzolino, Antonino Colloca, Alfonso Esposito, Maria Cristina Giugliano, Eleonora Cioli, Margaret Ottaviano, Lucia Iavarone, Renato Franco, Camila Scharf, Anna Russo, Salvatore Cappabianca, Paolo Antonio Ascierto, Valerio Nardone, Luigi Formisano, Stefania Napolitano, Davide Ciardiello, Fortunato Ciardiello, Teresa Troiani, Vincenzo De Falco

**Affiliations:** 1 Department of Precision Medicine, Università degli Studi della Campania “Luigi Vanvitelli”, Napoli, Italy; 2 Dermatology Unit, Department of Mental and Physical Health and Preventive Medicine, University of Campania “Luigi Vanvitelli”, Naples, Italy; 3 Clinical Medicine and Surgery, University of Naples Federico II, Naples, Italy; 4 Unit of Melanoma, Cancer Immunotherapy and Development Therapeutics, Istituto Nazionale Tumori IRCCS Fondazione Pascale, Naples, Italy; 5 Oncology Unit, Oncology Department, “Azienda Ospedaliero Universitaria (AOU) Dulbecco” Hospital, Catanzaro, Italy; 6 Unit of Medical Oncology, “Mons. A.R. Dimiccoli” Hospital, Barletta, Italy; 7 Medical Oncology Unit, Azienda Ospedaliera di Rilievo Nazionale (AORN) San Pio, Benevento, Italy; 8 PhD Course in Public Health, Department of Experimental Medicine, University of Campania “Luigi Vanvitelli”, Naples, Italy; 9 Pathology Unit, Department of Mental and Physical Health and Preventive Medicine, University of Campania “Luigi Vanvitelli”, Naples, Italy; 10 Division of Gastrointestinal Medical Oncology and Neuroendocrine Tumors, European Institute of Oncology, Istituto Europeo di Oncologia (IEO), IRCCS, Milano, Italy

**Keywords:** immunotherapy, ipilimumab, melanoma, nivolumab, real-world

## Abstract

**Introduction:**

Metastatic melanoma has historically had a poor prognosis; however, survival has improved with immunotherapies, such as PD−1 and CTLA−4 inhibitors, and molecular targeted therapies for BRAF−mutant tumors. The combination of nivolumab and ipilimumab is highly effective, though with increased rates of toxicity. In Italy, since January 2022, such therapeutic combination has been approved and reimbursed only for metastatic melanoma patients with brain metastases or with PD−L1 expression <1%.

**Methods:**

We conducted a real-world study in six academic centers in southern Italy and analyzed the efficacy and toxicity outcomes in 72 patients.

**Results:**

The response rate was 54% (39/72) and, after 13.6 months of median follow-up, a longer median progression-free survival [17.03 months (95% CI 4.8-18.6)] compared to the pivotal CheckMate-067 trial or to other real-world studies. Better survival correlated with objective responses (p<0.0001) and low disease burden (less than three metastatic sites) (p=0.0415). All patients achieving an objective response had tumors with high or intermediatetumor mutational burden. The rates of immune-related adverse events were similar to those reported in the literature, but there was a lower therapy discontinuation rate. This might be due to more appropriate managing of emerging immunotherapy toxicities.

## Introduction

Melanoma is a malignant tumor that arises from melanocytes. Its incidence has been steadily increasing over the past few decades, particularly in fair-skinned populations. According to GLOBOCAN, there were 330,000 new cases in 2022 and 58,600 deaths (1). Melanoma is known for its aggressive nature and potential for rapid metastatic spreading. Prognosis for metastatic melanoma has historically been poor, with five-year survival rates of only 15-20%. However, advancements in treatment options have significantly improved outcomes for patients with advanced disease. Current treatment modalities for metastatic melanoma include targeted therapies and immunotherapies. Targeted therapies have revolutionized the treatment landscape for patients with *BRAF*-mutated melanoma (approximately 40-60% of patients) leading to significant tumor regression in over 60% of patients (2, 3). However, most patients develop secondary resistance to these therapies and about 50% progress within a year (4). On the other hand, among the immunotherapeutic agents, anti-programmed death-1 (PD-1) inhibitors such as nivolumab and pembrolizumab have emerged as pivotal options. These monoclonal antibodies (mAbs) work by blocking an inhibitory pathway, thereby enhancing the immune system’s ability to recognize and attack melanoma cells (5). Clinical trials have demonstrated that both nivolumab and pembrolizumab can lead to durable responses in a subset of patients (6, 7). Additionally, ipilimumab, an anti-cytotoxic T lymphocyte antigen-4 (CTLA-4) monoclonal antibody, has shown efficacy in treating melanoma by further stimulating the immune response (8). The combination of nivolumab and ipilimumab appears to lead to higher response rates (RR) compared to monotherapy, albeit with an increased risk of immune-related adverse events. The CheckMate 067 trial highlighted this combination’s effectiveness, showing improved overall survival rates compared to either agent alone (9). Recently, on January 2022, the Italian Medicines Agency (AIFA) approved and reimbursed the combination of nivolumab plus ipilimumab for the treatment of metastatic melanoma only in adults with asymptomatic brain metastases or with PD-L1 expression < 1%, since in these two subgroups the combination has demonstrated a particular advantage compared to nivolumab alone. The main objective of this study is to evaluate the safety and efficacy of nivolumab plus ipilimumab in this subgroup of patients, in a real-world setting.

## Patients and methods

We retrospectively investigated clinical data coming from patients with metastatic melanoma treated with nivolumab plus ipilimumab (COMBO) in six centers in Southern Italy, within the Gruppo Oncologico dell’Italia Meridionale (GOIM) network.

Patients with the following characteristics were included in analysis: adults (> 18 years) with histologically confirmed unresectable distant metastases. Patients without brain metastases had PD-L1 expression of <1% in their biopsied sample Patients had at least one cycle of the standard dosing combination of ipilimumab 3 mg/kg and nivolumab 1 mg/kg every 3 weeks, followed by maintenance with nivolumab 3 mg/kg every 2 weeks or nivolumab 480 mg every 4 weeks. The data were recorded from January 2022 to December 2024. Patients with concomitant active malignancies, except for non-melanoma skin cancers or tumors *in situ* or benign neoplasms, were excluded.

All patient data were recorded in an internal computer database. Data included demographics, clinical and pathological information, as well as tumor responses and survival. The study protocol was approved by the Institutional Review Board of the University of Campania and was conducted in compliance with the Declaration of Helsinki and Good Clinical Practice (GCP) guidelines. All patients provided written informed consent authorizing the use of anonymized data for research purposes. Tumor responses were assessed based RECIST 1.1 criteria. Radiological evaluation was performed as in clinical practice every 2–4 months. Data regarding adverse events were collected and graded based on the National Cancer Institute Common Terminology Criteria for Adverse Events (NCI CTCAE), version 5.0. Survival curves were estimated using the Kaplan-Meier method. Statistical significance of survival curves was calculated using the Log-rank test. MedCalc (version 23.1.3) was used to generate survival curves and to calculate statistics throughout the entire manuscript.

The Foundation One CDx (F1CDx) assay was used for comprehensive genomic profiling of 13 biopsy samples (10). These patients belong to a single center participating in the study and are the only ones for whom material was available for genomic analysis.

## Results

### Patient characteristics

Overall, 72 patients treated with ipilimumab plus nivolumab combination from January 2022 to December 2024 met the inclusion criteria and were included in the study population. Baseline clinicopathological characteristics were reported in **Table 1**. Median age at the time of treatment initiation was 56.8 years with more males than females (63.9% vs 36.1%). The majority of cases were melanoma located on the trunk (55.5%) and on the arms (19.4%), including three acral melanoma. In 16.7% of cases the location of the primary lesion was unknown. More than half of the patients (63.9%) had a *BRAF^V600E^* mutation, while 79.2% had negative PD-L1 expression. The majority of patients (83.3%) received the COMBO as first-line therapy; 24% of patients were treated with COMBO after adjuvant therapy relapse (8.3% received adjuvant anti-PD1). Approximately one-third of patients had M1c disease, most of them having liver metastases, while 40.3% of patients had brain metastases.

**Table 1:** Patients characteristics.

Characteristics	All patients N = 72
Age (median, range)	56 (23-78)
Male (%)	46 (63.9%)
Melanoma subtype (%)	
Cutaneous	59 (82%)
Mucosal	1 (1.3%)
Unknown primary	12 (16.7%)
Disease at diagnosis	
Advanced upfront	28 (38%)
Recurrent disease	44 (62%)
BRAF status	
BRAF wild-type	26 (36.1%)
BRAF V600 mutant	46 (63.9%)
Metastasis at baseline	
Brain	
Yes	29 (40.3%)
No	43 (59.7%)
Liver	
Yes	20 (27.8%)
No	52 (72.2%)
Number of disease sites	
≥3	31 (43.1%)
<3	41 (56.9%)
ECOG PS (%)	
0	58 (80.5%)
1	11 (15.2%)
≥2	2 (2.7%)
Previous systemic treatments	
Treatment naïve	45 (62.5%)
Previously treated with immunotherapy	4 (5.6%)
Previously treated with BRAF inhibitors	21 (29.2%)
Both	2 (2.8%)
Combo administrated as:	
First line	60 (83.3%)
Second line	12 (16.7%)
N. of cycles with combo	
All four	34 (47.2%)
Two or three	20 (27.7%)
Only one	5 (6.9%)

### Efficacy outcomes

Objective response rate (ORR) was 54.2%, including 25% of complete responses (CR) (**Table 2**). Response rate was higher in patients without any previous systemic treatment (62.2%). Median follow-up time, estimated using the reverse Kaplan–Meier method, was 20.5 months. Among patients alive at last follow-up, the median follow-up was 11.9 months, while the median time to death among deceased patients was 6.2 months. At data cut-off, 47.2% of patients had disease progression. Four cycles of the combination therapy regimen were completed in 48.6% of patients. In 29.2% of patients, treatment is currently ongoing. During treatment, ten patients underwent local-regional treatments for palliative purposes (radiotherapy, surgery, or electro-chemotherapy).

**Table 2:** Survival and response outcome for the overall population and subgroups.

Population	mPFS (months)	p	CR	PR	SD	PD
All patients	17.03	/	18 (25%)	21 (29.2%)	20 (27.8%)	13 (18%)
First line (n=60)	18.64	0.0024	17 (28.3%)	19 (31.7%)	16 (26.6%)	8 (13.3%)
Second line (n=12)	1.87		1 (8.3%)	2 (16.6%)	4 (33.3%)	5 (41.7%)
BRAF mut (n=46)	15.65	0.5997	13 (28.3%)	13 (28.3%)	13 (28.3%)	7 (15.2%)
BRAF wild type (n=26)	18.64		5 (19.2%)	8 (30.8%)	7 (26.9%)	6 (23.1%)
With G3-G4 adverse events (n=27)	18.64	0.2079	9 (33.3%)	6 (22.2%)	8 (29.6%)	4 (14.8%)
No G3-G4 adverse events (n=45)	8.45		9 (20%)	15 (33.3%)	12 (26.7%)	9 (20%)
With brain metastases (n=29)	13.91	0.3338	8 (27.6%)	5 (17.2%)	11 (37.9%)	5 (17.2%)
No brain metastases (n=43)	17.03		10 (23.3%)	16 (37.2%)	9 (20.9%)	8 (18.6%)

Response rates. PFS, Progression Free Survival; OS, Overall Survival; CR, Complete Response; PR, Partial Response; SD, Stable Disease; PD, Progressive Disease.

**Figure 1:**
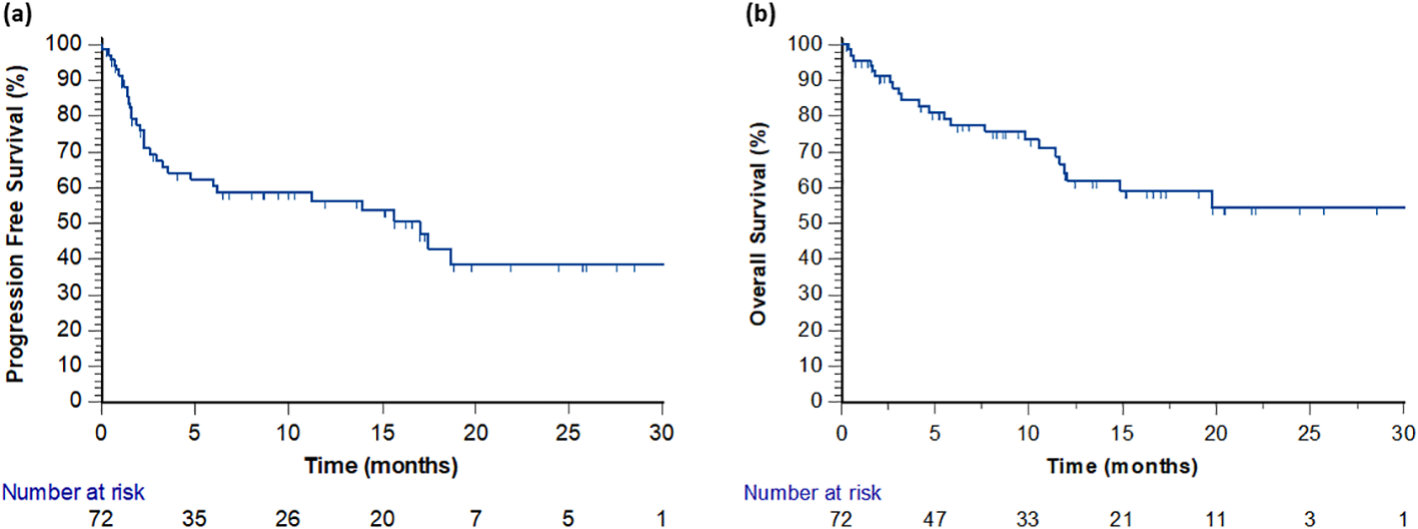
**(a)** Kaplan-Meier curves for progression free survival in the overall cohort. Censored patients: 17 pts after 12 months, 28 pts after 24 months. **(b)** Kaplan-Meier curves for overall survival in the overall cohort. Censored patients: 24 pts after 12 months, 41 pts after 24 months.

**Figure 2:**
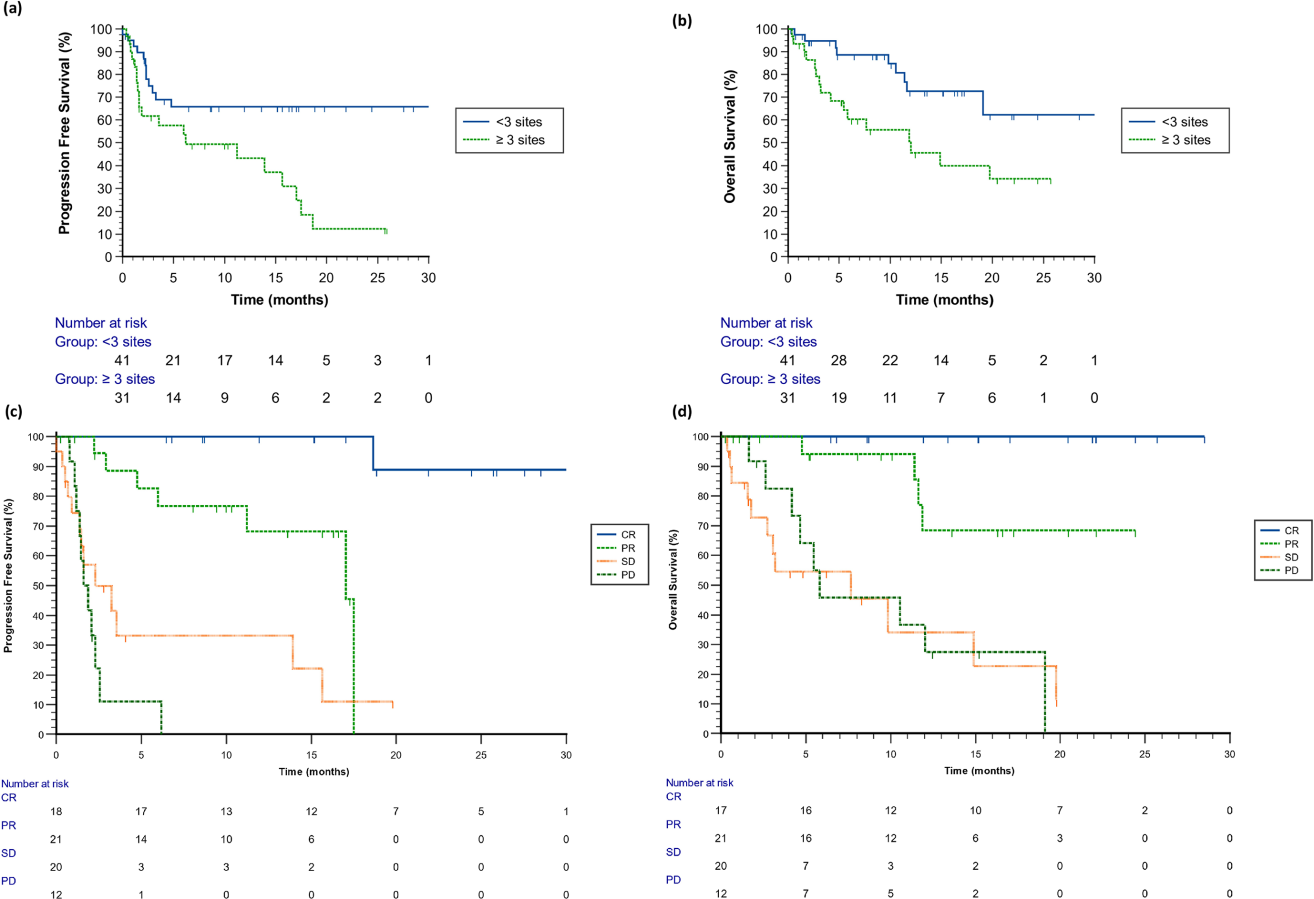
**(a)** Kaplan-Meier estimates for progression free survival according to number of metastatic sites. Censored patients in the <3 sites group: 13 pts after 12 months, 26 pts after 24 months. Censored patients in the ≥3 sites group: 4 pts after 12 months, 9 pts after 24 months. **(b)** Kaplan-Meier estimates for overall survival according to number of metastatic sites. Censored patients in the <3 sites group: 13 pts after 12 months, 30 pts after 24 months. Censored patients in the ≥3 sites group: 6 pts after 12 months, 14 pts after 24 months. Kaplan-Meier curves for progression free survival **(c)** and overall survival **(d)** according to best response.

Median PFS for the study cohort was 17.03 months (95% Confidence Interval (CI), 4.8-18.6 months) (**Figure 1a**). Median OS was not reached (**Figure 1b**). Of note, mPFS and RR were significantly better in patients who received first-line treatment than in those who received second-line treatment, even though the latter group consisted of only 12 patients (**Supplementary Data Sheet 1**). Survival correlated with response to therapy: median PFS in patients with CR, PR, SD and PD were not reached, 17.0, 2.3, 1.6 months, respectively (**Figure 2**). Of note, once excluding non-responder, that had a very poor prognosis, the Hazard Ratio (HR) for progression or death for patients with PR was 17.9 (95% CI 3.6-88.3; p=0.0004) compared to patients who had a CR. Similarly, median OS in patients with CR, PR, SD and PD were not reached, not reached, 7.7, 5.8 months, respectively. In the subgroup of patients who experienced PD as best response, no distinctive or univocal clinical characteristics emerged. Half of the cohort harbored a BRAF mutation. Only 4 of 12 patients had received prior adjuvant therapy, and 5 of 12 had been treated with first−line BRAFi+MEKi. Brain metastases were present in 5 of 12 patients, and 50% of the population had involvement of three or more metastatic sites. Cox regression analysis was also performed including as variables *BRAF* status (wild type vs mutant), treatment line (first- vs second-line), previous treatments in any setting (naive vs pre-treated), number of metastatic sites (<3 vs ≥3), presence of liver and/or brain metastases (yes vs no), objective response to therapy (yes vs no) and use of steroids during treatment (yes vs no). The analysis showed association with survival only with ORR (p<0.0001) and for the number of metastatic sites (p=0.0415). Only 37.5% of patients who progressed underwent further treatments, almost all with targeted therapy and only 2 with chemotherapy with poor efficacy.

### Safety

Treatment-related toxicity was experienced by72% of patients (**Table 3**). The most common side effects were diarrhea (37.5%), increased levels of alanine aminotransferase and aspartate aminotransferase (30.5%), skin toxicity (25%) and thyroiditis (19.4%). G3-G4 toxicity was reported in 28 patients (38.9%). Temporary suspension of treatment was required due to toxicity in 45.8% of patients, while permanent discontinuation occurred in 30.5% of patients. Corticosteroids were used to manage toxicities in 48.6% of patients; while 30.6% of patients needed hospitalization to manage toxicity, in almost every case for a time less than 2 weeks. Among the rare side effects, one patient experienced grade 3 thrombocytopenia and another patient had optical nerve neuropathy that caused loss of vision. Both patients were refractory to prednisone treatment and required a subsequent drug discontinuation after only 2 cycles. No toxicity-related deaths were recorded.

**Table 3:** Adverse events.

Adverse events	Any grade	G3-G4
Any adverse event	52 (72%)	28 (38.9%%)
Diarrhea	27 (37.5%)	8 (11.1%)
Cutaneous	18 (25%)	4 (5.6%)
Increase in alanine aminotransferase level	22 (30.5%)	6 (8.3%)
Increase in aspartate aminotransferase level	22 (30.5%)	5 (6.9%)
Hypothyroidism	14 (19.4%)	0 (0%)
Headache	5 (6.9%)	4 (5.6%)
Dyspnea	3 (4.1%)	1 (1.4%)
Treatment-related adverse events leading to suspension	32 (45.8%)	
Treatment-related adverse events leading to discontinuation	22 (30.5%)	
Patient needed use of corticosteroids	35(48.6%)	

### Molecular findings

**Figure 3:**
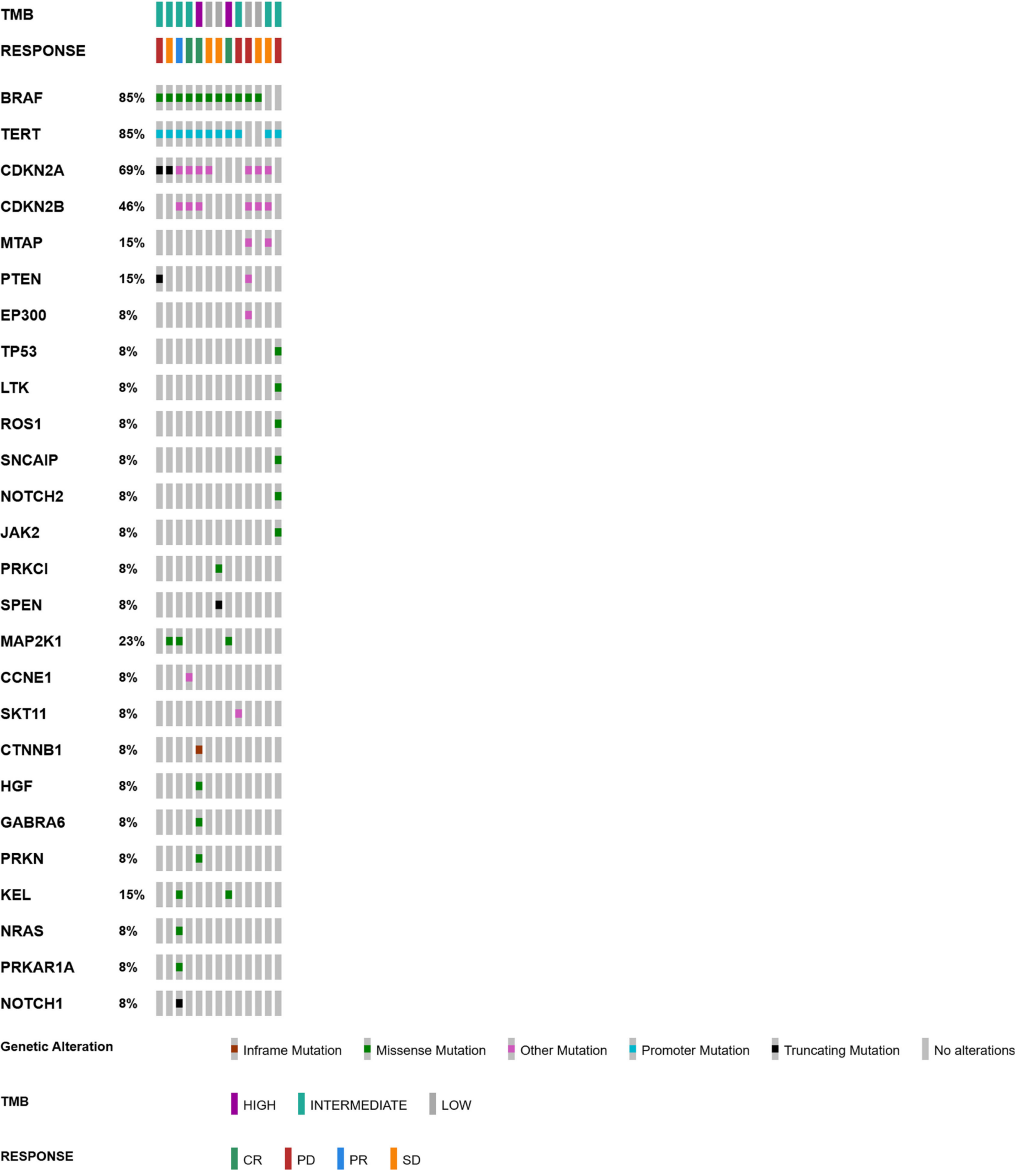
Oncoprint scheme representing genetic alterations, tumor mutational burden (TMB) and objective response of 13 patients.

Tissues samples from primary melanoma were evaluable and evaluated by next generation sequencing (Foundation One CDx) for comprehensive genomic profiling (CGP) (**Figure 3**). About 46% of patients had both CDKN2A and CDKN2B gene loss. In 85% of the samples *TERT* promoter region was mutated. In one patient, homologous recombination deficiency (HRD) was found. We next evaluated if tumor mutational burden (TMB) correlated to treatment response. TMB was defined as TMB-High, TMB-Intermediate, or TMB-Low as described by Palmeri et al. (11) Obiective responses (CR and PR) were observed in patients whose tumors were classified aTMB-High or TMB-intermediate (**Figure 3**).

## Discussion

The therapeutic use of nivolumab in combination with ipilimumab has dramatically changed the clinical landscape for patients with metastatic melanoma. The Checkmate 067 study has shown that approximately 50% of patients treated with this combination survive longer than 10 years (9). However, this therapeutic regimen is currently approved only for patients with brain metastases or with immune histochemical expression of PD-L1 less than 1%, since these patients had the greater benefit from the combination. There are so far a few real-world studies on this subgroup of patients. Therefore, we aimed to collect safety and efficacy data in six oncology centers in southern Italy that have administered this treatment in this setting. The results reported here confirmed the efficacy in terms of response rate and survival. Comparison with the pivotal study (9) and with other real-life studies (11) shows a similar response rate with better mPFS and mOS. In fact, mPFS reached 17.03 months versus 11.5 months reported in the pivotal study or 6.6 months in the Dutch study (12). Currently, mOS has not been reached, in comparison with 71.9 months for Checkmate 067 study and 27.8 months for the van Zeijl et al. study, respectively (12). The better survival results reported here could be due to the prevalence of prognostic positive factors, such as the presence in more than 50% of them of less than 3 metastatic sites and the excellent performance status at baseline, although there was also a higher percentage of other potentially negative prognostic factors, such as *BRAF* mutations (63.9%) or brain metastases (40%). These parameters were analyzed to assess the association with improved survival, but the results were not significant. The association with adverse events also showed no correlation with survival (**Supplementary Data Sheet 1**). Cox regression analysis confirmed the association between outcome and disease burden, as well as with best response to treatment. Unlike other studies (13), we initially found a correlation between survival and treatment line (first- versus second-line), but the Cox analysis did not confirm this, probably because the number of patients included in this study who received doublet as a second-line treatment was relatively small (12). This study also reports the molecular data for CGP of a subgroup of 13 patients. Even with the limitations due to the small number, the association between objective responses and TMB-high or TMB-intermediate occurred in all patients and is in agreement with the results reported by Hodi et al. (14) The percentage of patients with grade 3–4 adverse events (38.9%) recorded in our study was significantly lower than that in published clinical trials, where it consistently exceeds 40-50% (9, 12, 13). There was also a lower than expected therapy discontinuation rate. In our opinion, these data, despite being a retrospective study, do not reflect underreporting of safety data, precisely because they concern severe adverse events, which are therefore unlikely to be missed in the medical records of these patients. The most likely cause is the fact that currently there is greater knowledge and better expertise in recognizing and managing the side effects of this treatment compared to studies conducted several years ago.

In conclusion, treatment of metastatic melanoma remains an unmet need for more than half of patients. The use of combination strategies (15) or promising sequences (16) have not yet demonstrated superior efficacy to the combination of nivolumab and ipilimumab. However, administering this treatment in earlier settings (neoadjuvant) (17) raises the question of what to do when the disease progresses. Furthermore, the recent approval of the combination of nivolumab and relatlimab (18) has added another piece to the puzzle, but there are no studies comparing the two combinations in the first-line setting. In any case, this study confirmed the efficacy and tolerability of nivolumab plus ipilimumab in real-world settings, including the subgroup of patients for whom the combination is reimbursed in Italy by the Public Health Service.

## Data Availability

The raw data supporting the conclusions of this article will be made available by the authors, without undue reservation.
