# Supplementary material for: Clinical activity and safety of nivolumab in combination with ipilimumab in metastatic melanoma: findings from REALIPINIVO, a real-world study
Source: Front Immunol. 2026 Apr 16;17:1738772. doi: 10.3389/fimmu.2026.1738772 (PMC13128669; doi:10.3389/fimmu.2026.1738772)
Supplement: Supplementary file 1 [file DataSheet1.docx]

**SUPPLEMENTARY DATA**

| **Hazard ratios** | **Variable** | **Estimate** | **95% CI (profile likelihood)** |  |
| --- | --- | --- | --- | --- |
| Exp(β1) | BRAF_status[WT] | 1,026 | 0,3872 to 2,653 |  |
| Exp(β2) | Line[First] | 0,5909 | 0,1393 to 2,236 |  |
| Exp(β3) | Treatment_Naive[Yes] | 2,531 | 0,8313 to 9,252 |  |
| Exp(β4) | Number_of_metastatic_sites[<3] | 0,3496 | 0,1239 to 0,9505 |  |
| Exp(β5) | Liver_met[NO] | 1,71 | 0,6552 to 4,723 |  |
| Exp(β6) | Brain_met[NO] | 1,207 | 0,5209 to 2,849 |  |
| Exp(β7) | R_vs_NR[R] | 0,05948 | 0,01848 to 0,1668 |  |
| Exp(β8) | Steroid_therapy[no] | 2,088 | 0,8710 to 5,115 |  |
|  |  |  |  |  |
| **Sig. diff. than zero?** | **Variable** | **\|Z\|** | **P value** | **P value summary** |
| β1 | BRAF_status[WT] | 0,05291 | 0,9578 | ns |
| β2 | Line[First] | 0,7538 | 0,4509 | ns |
| β3 | Treatment_Naive[Yes] | 1,531 | 0,1257 | ns |
| β4 | Number_of_metastatic_sites[<3] | 2,038 | 0,0415 | * |
| β5 | Liver_met[NO] | 1,071 | 0,2841 | ns |
| β6 | Brain_met[NO] | 0,4362 | 0,6627 | ns |
| β7 | R_vs_NR[R] | 5,081 | <0,0001 | **** |
| β8 | Steroid_therapy[no] | 1,642 | 0,1005 | ns |

**Table S1.** Table of the Hazard ratios and 95%CIs for the features analyzed with Cox regression analysis


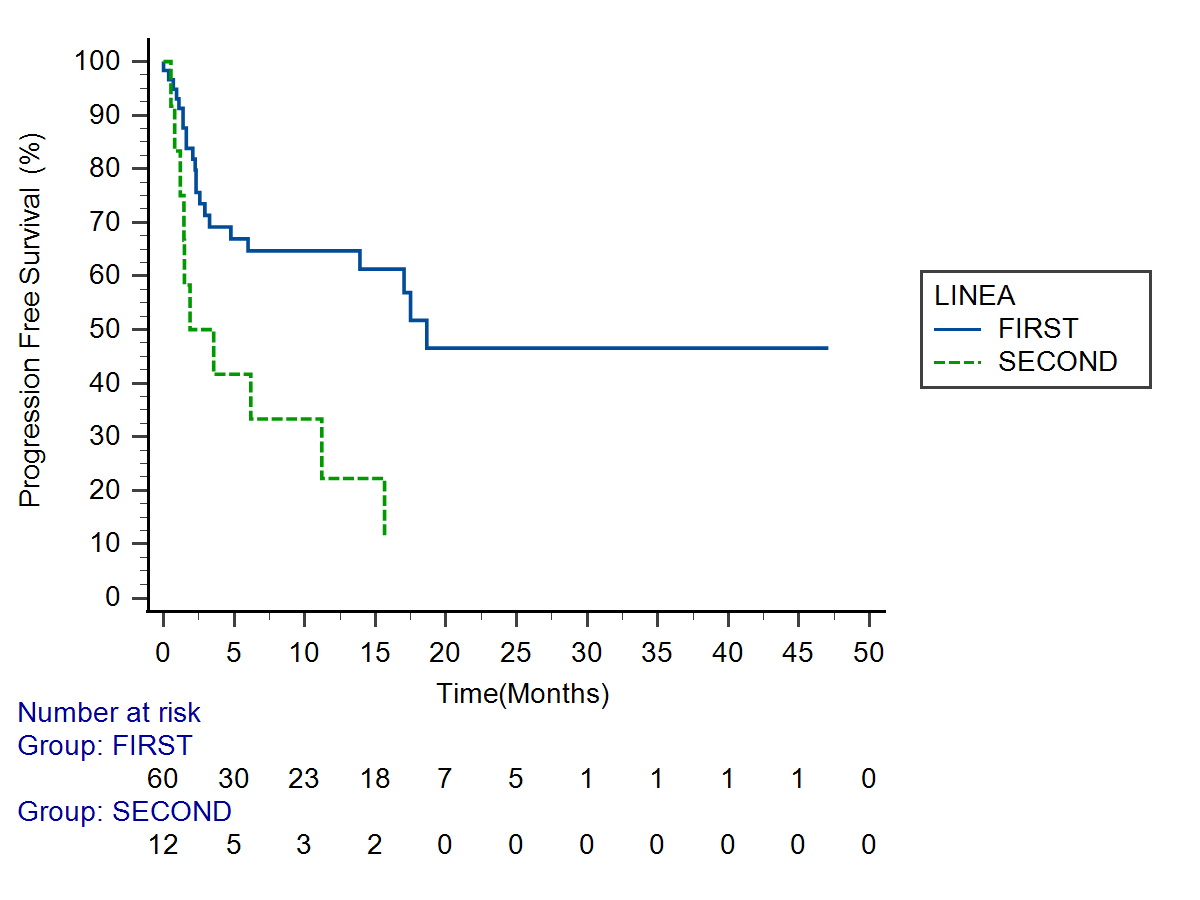


**FIG S1**

*The Kaplan Meier shows benefit in MM treated in first line with the IO combo(p=0.0024). The median PFS in first line group was 18.42 months vs 1.874 in second line group.*


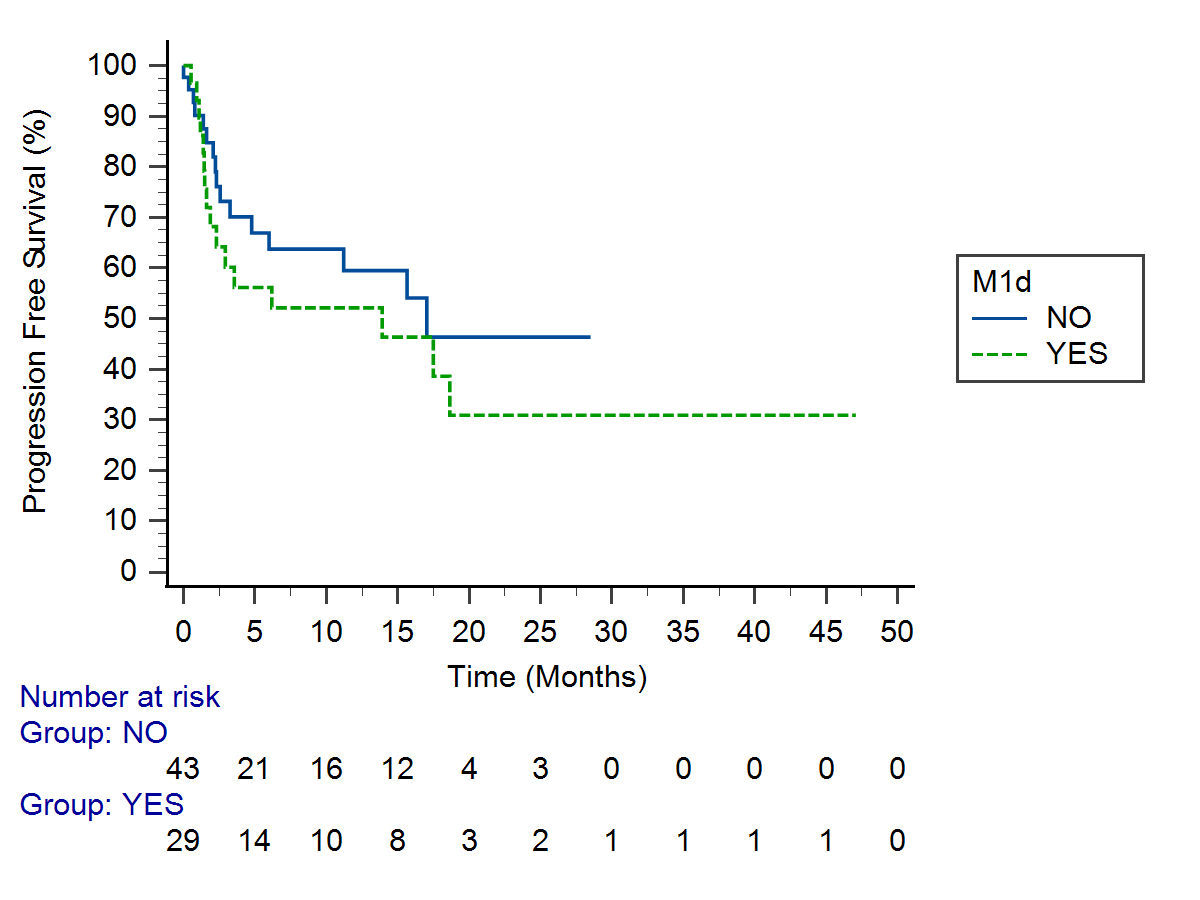


**FIG S2**

*The Kaplan Meier shows no significant difference between the subgroup with and without brain localization (p=0.3338).*


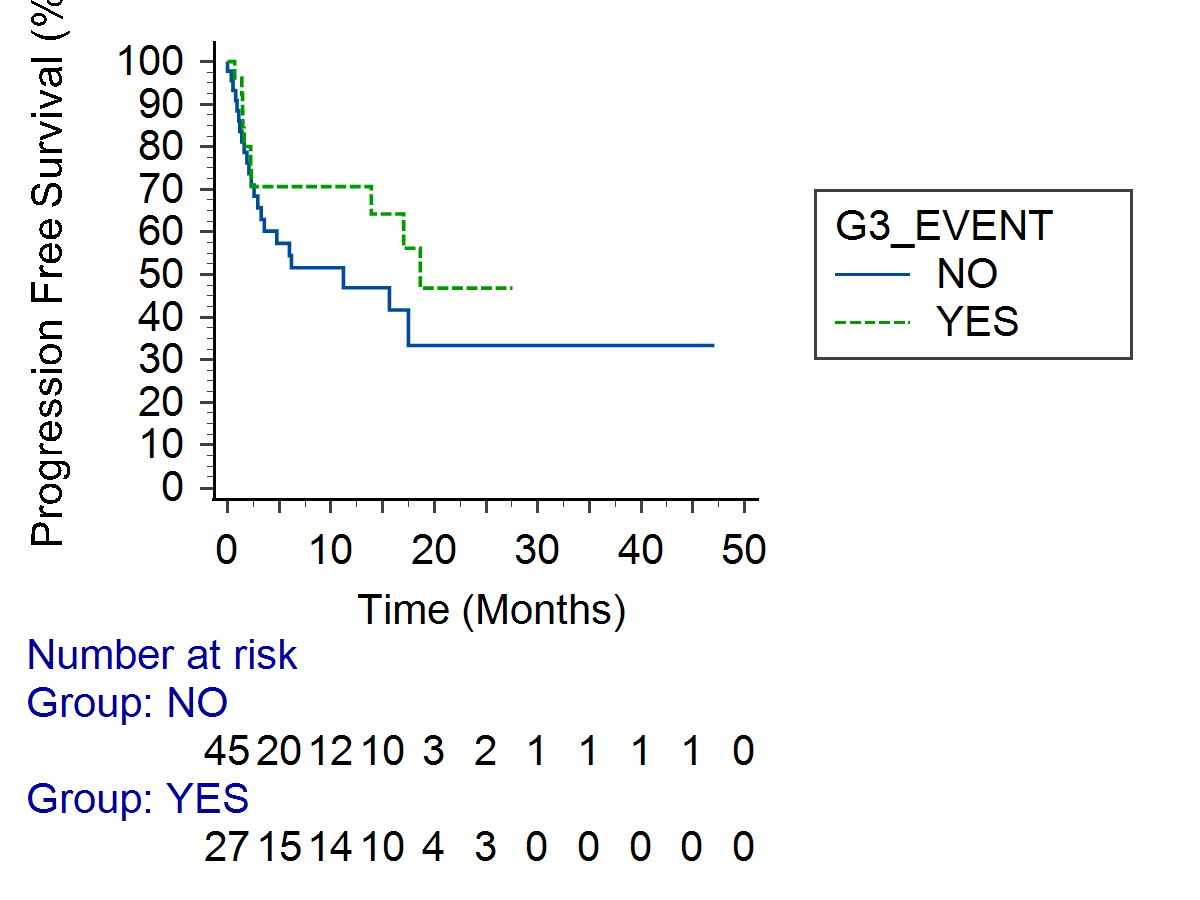


**FIG S3**

*The Kaplan Meier shows no significant difference between the subgroup with and without G3-G4 ADR events (p=0.2079).*


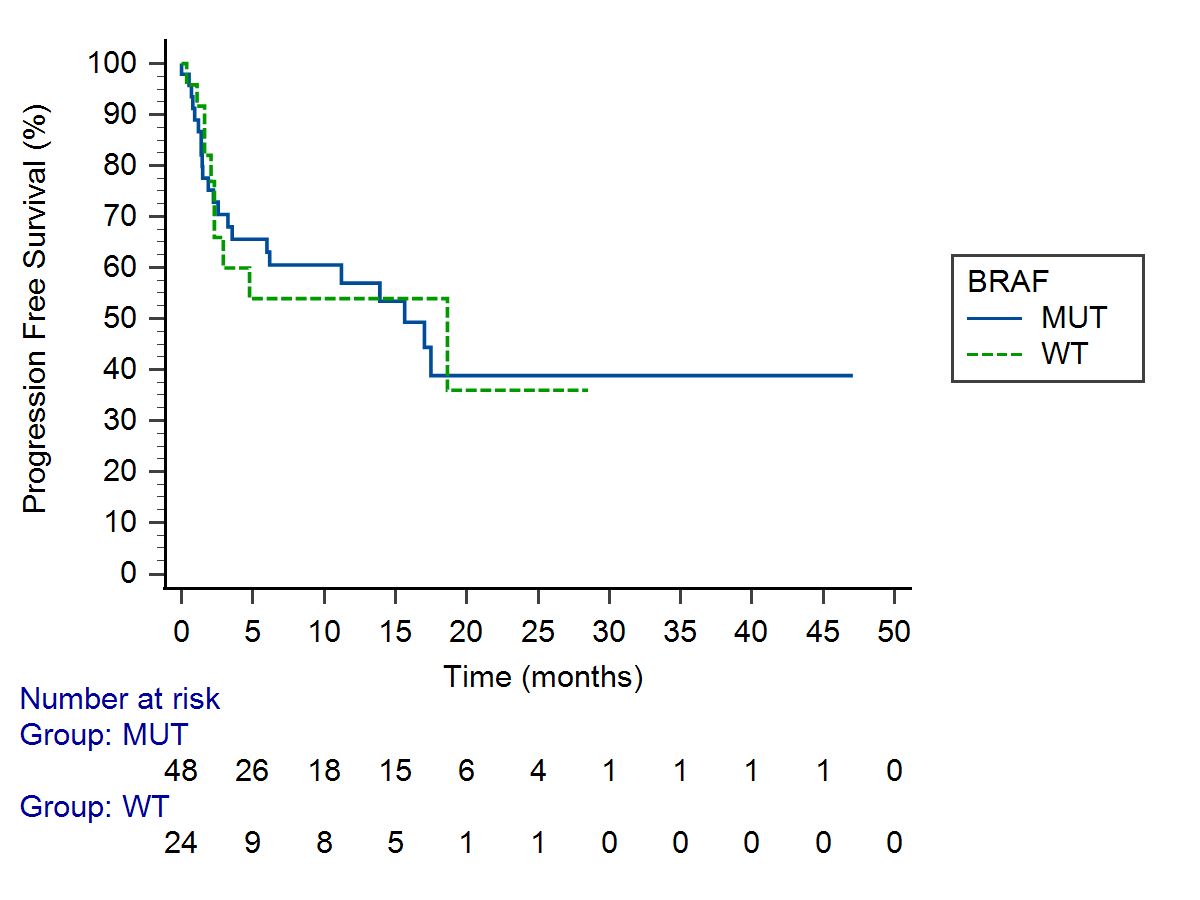


**FIG S4**

*The Kaplan Meier shows no significant difference between the subgroup with and without BRAF mutation (p=0.9539).*


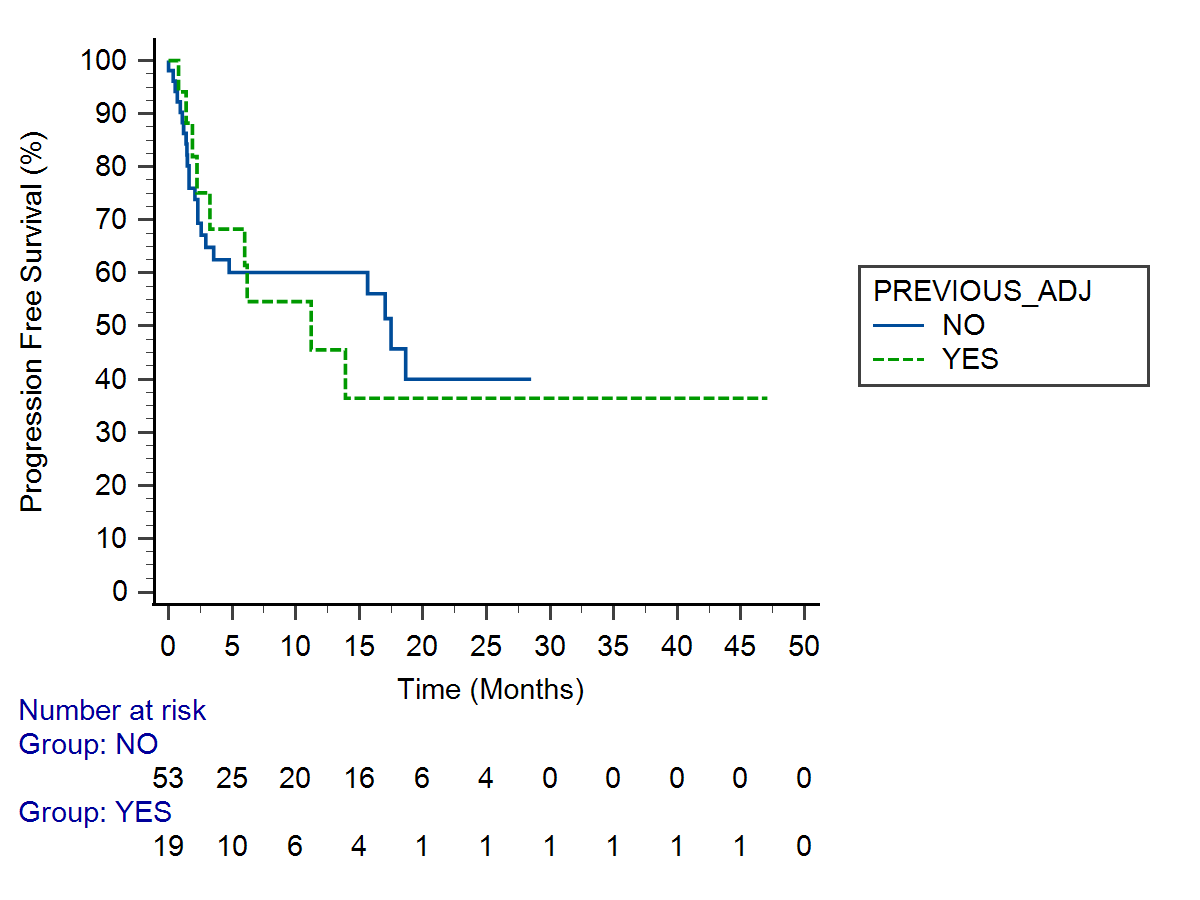


**FIG S5**

*The Kaplan Meier shows no significant difference between the subgroups previously treated in adjuvant regimens (p=0.7306).*
